# Non-Targeted Metabolomic Analysis of Methanolic Extracts of Wild-Simulated and Field-Grown American Ginseng

**DOI:** 10.3390/molecules24061053

**Published:** 2019-03-18

**Authors:** Hongqiang Lin, Hailin Zhu, Jing Tan, Han Wang, Qinghai Dong, Fulin Wu, Yunhe Liu, Pingya Li, Jinping Liu

**Affiliations:** Research Center of Natural Drug, School of Pharmaceutical Sciences, Jilin University, Changchun 130021, China; linhq17@mails.jlu.edu.cn (H.L.); 13578965875@163.com (H.Z.); tanjing17@mails.jlu.edu.cn (J.T.); hanw17@mails.jlu.edu.cn (H.W.); dongqh17@mails.jlu.edu.cn (Q.D.); wufl17@mails.jlu.edu.cn (F.W.); lyh133700@163.com (Y.L.)

**Keywords:** wild-simulated American ginseng, field-grown American ginseng, screening analysis, metabolomic analysis

## Abstract

Aiming at revealing the structural diversity of secondary metabolites and the different patterns in wild-simulated American ginseng (WsAG) and field-grown American ginseng (FgAG), a comprehensive and unique phytochemical profile study was carried out. In the screening analysis, a total of 121 shared compounds were characterized in FgAG and WsAG, respectively. The results showed that both of these two kinds of American ginseng were rich in natural components, and were similar in terms of the kinds of compound they contained. Furthermore, in non-targeted metabolomic analysis, when taking the contents of the constituents into account, it was found that there indeed existed quite a difference between FgAG and WsAG, and 22 robust known biomarkers enabling the differentiation were discovered. For WsAG, there were 12 potential biomarkers including two ocotillol-type saponins, two steroids, six damarane-type saponins, one oleanane-type saponins and one other compound. On the other hand, for FgAG, there were 10 potential biomarkers including two organic acids, six damarane-type saponins, one oleanane-type saponin, and one ursane. In a word, this study illustrated the similarities and differences between FgAG and WsAG, and provides a basis for explaining the effect of different growth environments on secondary metabolites.

## 1. Introduction

American ginseng (*Panax quinquefolius* L.) is grouped into four categories: wild, wild-simulated, woods-grown, and field-grown [1,2]. The herb growing in its native habitat is called wild American ginseng. Wild-simulated American ginseng (WsAG) refers to a method of growing ginseng in a hardwood forest environment under natural conditions without any other human intervention [2,3,4]. As such, WsAG roots are indeed indistinguishable from the wild roots due to the similar characteristics. When the seeds are planted in hardwood forests, and are grown in prepared rows or beds, or with removed ground vegetation or fertilizer and pesticides being available [5,6,7], it is called as wood-grown ginseng. This variety of American ginseng requires 6 to 9 years to mature before harvesting [8]. Different from wild-simulated category, the quality of wood-grown ginseng is between that of the wild and field-grown categories. That means, wood-grown American ginseng cannot be considered a substitute of the wild one. Field-grown American ginseng (FgAG), also called cultivated American ginseng, is intensely cultivated under artificial shade structures using fertilizers and pesticides [4,9]. Generally speaking, FgAG is harvested after 3–4 years, while WsAG is collected at least after 10–20 years or longer [10].

Modern pharmacological studies have shown that American ginseng has immunomodulatory [11], anti-tumor [12], anti-fatigue [13,14], anti-diabetic [15,16,17], anti-oxidant effects [18] and the functions of improving impaired memory and learning functions [14,19], etc. Furthermore, it is the traditional belief that roots from the wild are more medicinally efficacious, more potent and more valuable than those from cultivated sources, and wild roots thus command much higher prices on the Chinese medicine market [20,21,22]. But, since the late 18th century, natural wild American ginseng resources suffered a sharp decline due to predatory exploitation in North America under the influence of economic interests, and are nearing extinction now [23]. Meanwhile WsAG, with high quality and four to ten times the retail value of field-grown roots, is similar to the wild one [4]. Actually, planting wild-simulated ginseng is encouraged with the aim of reducing harvest pressure on wild populations [24]. Wild and wild-simulated roots could share the same export and trade regulations due to the similar morphology phenotype and market value [8,25]. Recently, because the so-called wild-simulated American ginseng could capitalize on the premium paid for wild-appearing roots, and the species appeared well suited to the practice of forest farming, American ginseng has been recommended as an agroforestry crop candidate [26]. With the continuous expansion of the folk and clinical applications of American ginseng, it is necessary to conduct an in-depth study on the chemical constituents of American ginseng aiming to clarify the material basis of efficacy. So far, there are a few comparative analysis on FgAG and wild American ginseng [27,28]. These results showed that ginsenosides are different between them [26], especially, the ratios of Rg_1_/Rd, Rg_1_/Rb_1_ and (Rg_1_ + Re)/Rd are characteristic markers for differentiating these two groups [28]. However, a comparative study on the chemical composition between FgAG and WsAG does not exist.

Untargeted metabolomics, being able to profile diverse classes of metabolites, has been successfully applied to compare and identify the overall small-molecule components of different groups of samples [29]. Ultra-high performance liquid chromatography (UPLC) combined with quadrupole time-of-flight tandem mass spectrometry (QTOF-MS) and multivariate statistical analyses are often applied to profile the different groups. For multivariate statistical analyses, principal component analysis (PCA) and orthogonal partial least squares discriminant analysis (OPLS-DA) are the most common statistical methods. Meanwhile, UPLC-QTOF-MS combined with the automated data processing software UNIFI was often applied recently in the characterization of chemical components of herbal medicines [30,31,32,33,34,35]. When the coeluting constituents possessed different *m*/*z* values, HR-MS can provide a specific and accurate mass. While, UNIFI, a highly comprehensive, high throughput, efficient and simple platform, offers a method for integrating data acquisition, data mining, library searching and reporting generation.

Aiming to find out the similarities and differences between FgAG and WsAG, and to provide a reference for quality control and material basis of efficacy, the screening and the comparative analysis of chemical constituents in FgAG and WsAG is conducted for the first time in this paper. The shared constituents would be evaluated with the UPLC-QTOF-MS method combined with UNIFI. The characteristic components were to be found using the untargeted metabolomics method. The results will also be helpful in explaining the different pharmacological activities and controlling the quality of FgAG and WsAG.

## 2. Results

### 2.1. Identification of Components from FgAG and WsAG Based on the UNIFI Platform

**Figure 1:**
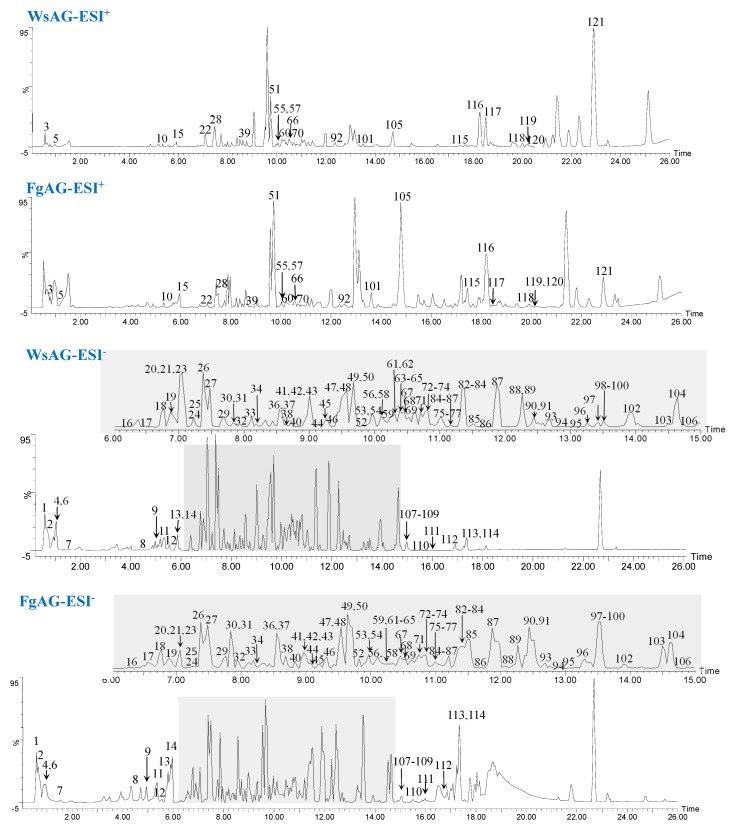
The representative base peak intensity (BPI) chromatograms of FgAG and WsAG in negative and positive modes.

**Figure 2:**
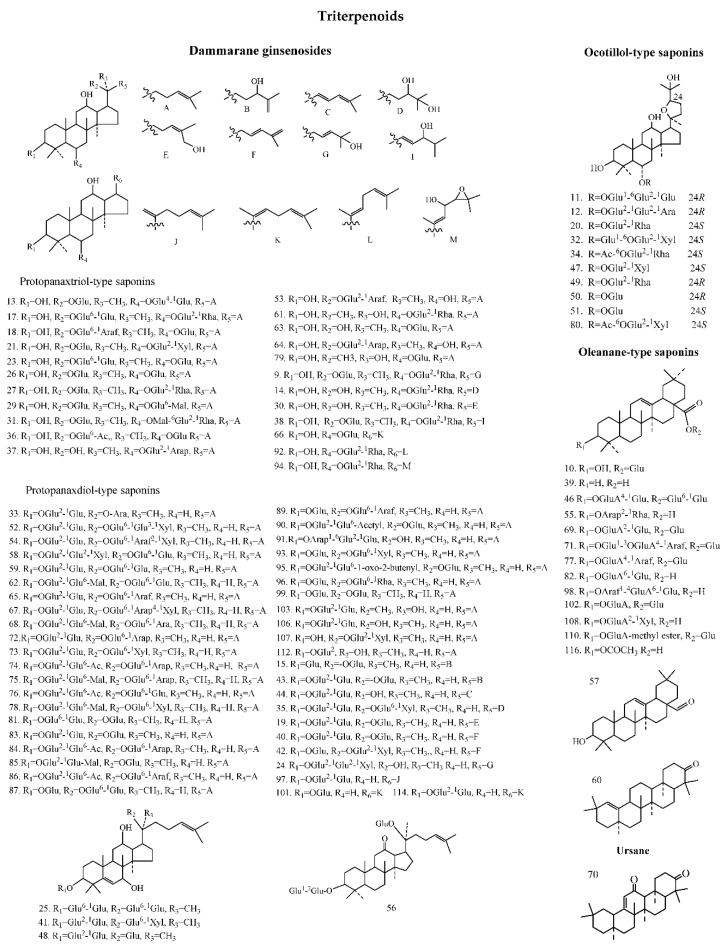

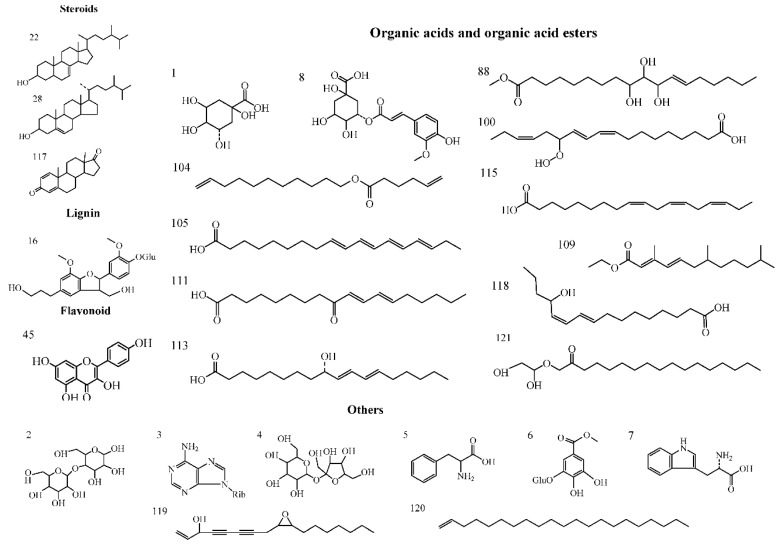
Chemical structures of compounds identified in FgAG and WsAG.

As a result of our screening analysis, a total of 121 compounds were identified or tentatively characterized in both positive and negative mode from FgAG and WsAG (Table 1), the base peak intensity (BPI) chromatograms are shown in Figure 1, and their chemical structures are shown in Figure 2.

In FgAG and WsAG, these compounds were all shared constituents, including 47 protopanaxdiol-type saponins, 23 protopanaxtriol-type saponins, 15 oleanane- type saponins, 10 ocotillol-type saponins, one ursane, one flavonoid, one lignin, 12 organic acids and organic acid esters, three steroids, and eight other compounds.

### 2.2. Biomarker Discovery for FgAG and WsAG

**Figure 3:**
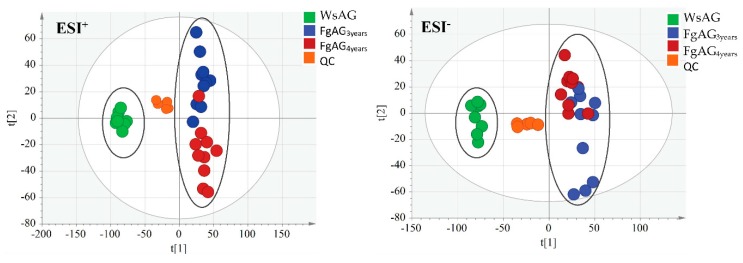
The PCA of FgAG and WsAG in positive mode and negative mode.

The QC injections were clustered tightly in PCA indicating a satisfactory stability of the system. The PCA 2D plots of the samples from FgAG and WsAG groups were classified into two clusters according to their common spectral characteristics (Figure 3), with the FgAG samples of different years clustered into one group, while the WsAG samples were clustered into another group. The FgAG and WsAG samples were clearly separated, indicating that these two herb species could be easily differentiated.

**Figure 4:**
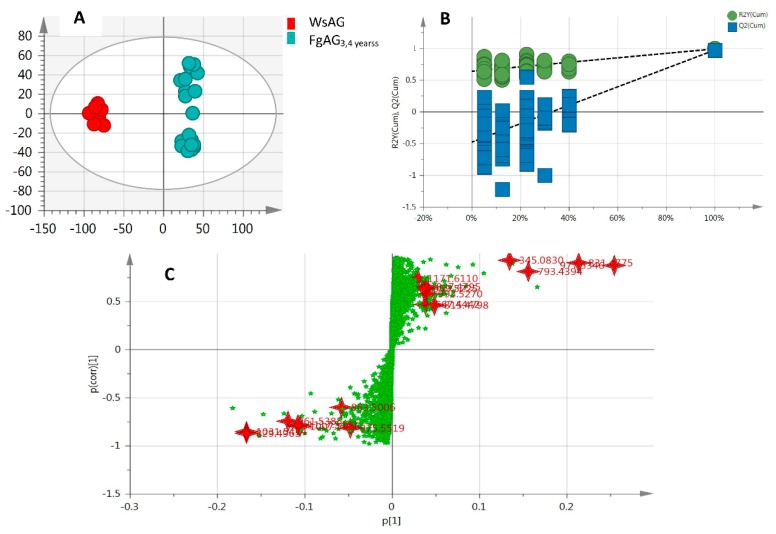
The OPLS-DA (**A**); permutation tests (**B**) and S-plot (**C**) in negative mode.

**Figure 5:**
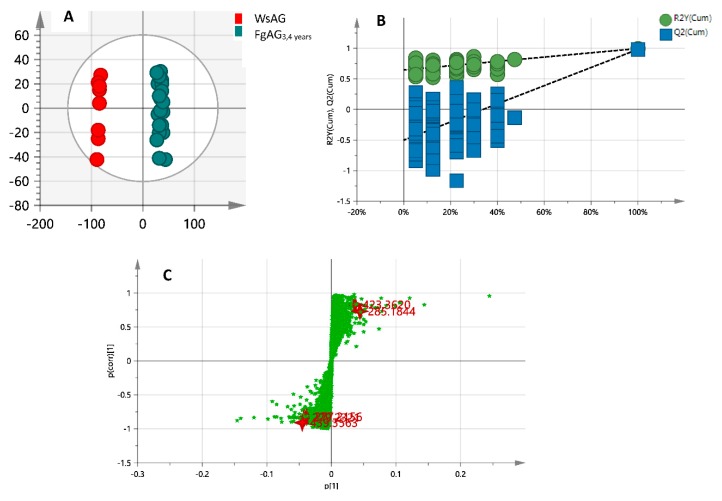
The OPLS-DA (**A**); permutation tests (**B**) and S-plot (**C**) in positive mode.

**Figure 6:**
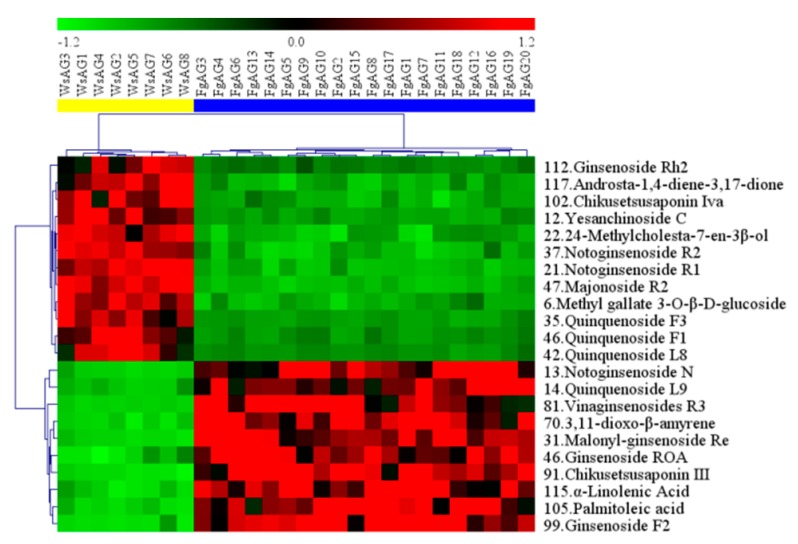
The heatmap visualizing the intensities of potential biomarkers.

After OPLS-DA plots (Figure 4A and Figure 5A) in both negative and positive modes were generated, the maximum separation between MsAG and FgAG groups was available. In the sufficient permutation test, the lines of grouping samples were significantly located underneath the random sampling lines (Figure 4B and Figure 5B), which indicated a fine validity for the following characteristic metabolites biomarkers identification [42]. S-plots were then created to explore the potential chemical markers that contributed to the differences. Based on *p* values (*p* < 0.05) and VIP values (VIP > 3) [30,61] from univariate statistical analysis, 22 robust known biomarkers enabling the differentiation between FgAG and WsAG, were marked and listed (Figure 4C and Figure 5C and Table 2). Additionally, a heatmap was generated from these biomarkers in order to systematically evaluate the biomarkers (Figure 6), which visually showed the intensities of potential biomarkers between two species.

## 3. Discussion

In the screening analysis, 121 compounds were characterized in FgAG and WsAG, respectively. The results showed that both of these kinds of American ginseng were rich in natural components. These 121 compounds were all shared constituents in FgAG and WsAG, which means that they were similar in terms of the kinds of compound they contained. It has been reported that there are high ginsenoside contents in American ginseng. In this study, ginsenosides were also the main chemical components. Besides the most common dammarane-type and oleanane-type saponins, the ocotillol-type saponins are also occupying a notable proportion. The ocotillol-type is the characteristic type of saponin enabling American ginseng to be differentiated from Asian ginseng. So far, the studies on the mechanism of biosynthesis were focused on dammarane-type and oleanane-type ginsenosides. For example, dammaranediol was obtained by DS (dammarenediol synthase), and then modified by CYP450 to obtain dammarane-type saponins. Another example, oleanane-type ginsenosides were obtained by modifing *β*-amyrin with CYP450 and UGT (UDP-glycosyltransferase). Actually, there were little literature about the mechanism of ocotillol-type ginsenoside biosynthesis. The phytochemicals in WsAG and FgAG might provide a material basis for mechanistic studies. In short, this comprehensive and unique phytochemical profile study revealed the structural diversity of secondary metabolites and the similar patterns in FgAG and WsAG.

Furthermore, in non-targeted metabolomic analysis, when taking the contents of the constituents into account, it was found that there indeed existed quite a few differences between FgAG and WsAG, and 22 robust known biomarkers enabling the differentiation were discovered. This study illustrated the differences between FgAG and WsAG, and provided a basis for explaining the effect of different growth environments on secondary metabolites. For WsAG, there are 12 potential biomarkers, including two ocotillol-type saponins (**12**, **47**), two steroids (**22**, **117**), six damarane-type saponins (**21**, **35**, **37**, **42**, **44**, **112**), one oleanane-type saponin (**102**) and one other compound (**6**). The contents of these 12 components in WsAG were much greater than in FgAG. On the other hand, for FgAG, there are 10 potential biomarkers including two organic acids (**105, 115**), six damarane-type saponins (**13, 14, 31, 46, 81, 91, 99**), one oleanane-type saponin (**46**), and one ursane (**70**), which contents in FgAG were much greater than in WsAG. It has been reported that wild American ginseng has better biological activity than the FgAG. As is known, biological activity is caused by the high contents of phytochemicals. Correlation studies between potential markers and biological activities could be performed in the future.

Even so, there are still several unresolved issues. For example, as shown in BPI chromatograms, though 121 compounds were identified, there are still some unidentified components. Further research should be carried on based on the formula of these unknown compounds.

## 4. Materials and Methods

### 4.1. Materials and Reagents

Twenty eight batches of commercially available FgAGs and WsAGs root products were collected or purchased from different cultivation areas in China and American, including 20 batches of FgAGs and eight batches of WsAGs. A detailed sample list is provided in Table 2.

For FgAGs, six roots of each sample were selected for analysis, while for WsAGs, 2–3 roots of each sample were analyzed. All the herbs were authenticated by the authors and the corresponding voucher specimens have been deposited in the Research Center of Natural Drug, School of Pharmaceutical Sciences, Jilin University, China.

A total of 25 saponins were isolated in our laboratory and identified by spectroscopic data. Among of these saponins, ginsenoside Ro [69], 15 ginsenosides [70,71] (Rb_1_, Rb_2_, Rb_3_, Rc, Rd, Re, 20(*S*)-Rg_3_, 20(*R*)-Rg_3_, 20(*S*)-Rh_2_, Rg_1_, 20(*R*)-Rg_2_, 20(*S*)-Rh_1_, 20(*R*)-Rh_1_, pseudo-ginsenoside F_11_, pseudo-ginsenoside RT_5_) and another six saponins [71] (quinquenoside L_8_, L_9_, L_11_, F_3_, 24(*S*)-pseudo- ginsenoside *F_11_*, gypenoside XVII) were isolated and identified by our group.

Oleanolic acid-28-*O*-*β*-d-glucopyranoside, ginsenoside Rs_1_, -Rs_2_ and methyl gallate-3-*O*-*β*-d-glucoside were also isolated in our laboratory and identified by NMR spectroscopy. Adenosine, α-maltose, l-tryptophan, notoginsenoside R_1_, kaempferol, l-phenylalanine, sucrose, palmitoleic acid, quinic acid and α-linolenic acid were purchased from Beijing Zhongke Quality Inspection Biotechnology Co., Ltd. (Beijing, China).

Acetonitrile and methanol suitable for UPLC-MS were purchased from Fisher Chemical Company (Geel, Belgium). Formic acid was purchased from Sigma-Aldrich Company (St. Louis, MO, USA). Deionized water was purified using a Millipore water purification system (Millipore, Billerica, MA, USA). All other chemicals were of analytical grade.

### 4.2. Sample Preparation and Extraction

All samples were respectively air-dried, grinded and sieved (Chinese National Standard Sieve No. 3, R40/3 series) to get a homogeneous powder. Then each fine powder was accurately weighed (0.2 g) and soaked with 10 mL of methanol overnight. On the second day, each powder was extracted in an ultrasonic bath (power of 250 W, frequency of 40 kHz) for half an hour. After cooling to room temperature, the lost weight was replenished with methanol. After filtering through a syringe filter (0.22 μm), the extracts were injected directly into the UPLC system. In addition, to ensure the stability and suitability consistency of MS analysis, a quality control (QC) sample was prepared by pooling the same volume (50 μL) from every sample and five QC injections were performed randomly through the whole worklist. The volumes injected for samples and QC were all 5 μL for each run.

### 4.3. UPLC-QTOF-MS

UPLC-QTOF-MS^E^ analysis was performed on a Waters Xevo G2-XS QTOF mass spectrometer (Waters Co., Milford, MA, USA) equipped with a UPLC system through an electrospray ionization (ESI) interface. Chromatographic separation was performed on an ACQUITY UPLC BEH C_18_ (100 mm × 2.1 mm, 1.7 μm) from Waters Corporation (Milford, MA, USA). The mobile phases were composed of eluent A (0.1% formic acid in water, *v*/*v*) and eluent B (0.1% formic acid in acetonitrile, *v*/*v*) with flow rate of 0.4 mL/min. The elution conditions applied were: 0→2 min, 10% B; 2→26 min, 10~100% B; 26→29 min, 100% B; 29→29.1 min, 100~10% B; 29.1→32 min, 10% B. The temperature of the autosampler and the UPLC column manager were set at 15 °C and 30 °C respectively. Mixtures of 90/10 and 10/90 water/acetonitrile were used as the weak wash solvent and the strong wash solvent respectively. The mass spectrum was acquired from 100 to 1500 Da in MS^E^ mode. The positive mode conditions were: capillary voltage, 2.6 kV; cone voltage, 40 V; source temperature, 150 °C; desolvation temperature, 400 °C; cone gas flow, 50 L/h; desolvation gas flow, 800 L/h. Negative mode conditions were identical to the positive mode conditions except for capillary voltage (2.2 kV). In MS^E^ mode, data acquisition was performed via the mass spectrometer by rapidly switching from a low-collision energy (CE) scan to a high-CE scan during a single LC run. The low energy function was set to 6 V, while ramp collision energy of high energy function was set to 20~40 V. Leucine enkephalin (*m*/*z* 556.2771 in positive mode and 554.2615 in negative mode) was used as external reference of Lock Spray™ infused at a constant flow of 10 μL/min. During acquisition, data were collected in continumn mode for the screening analysis, and in centroid mode for the metabolomics analysis.

### 4.4. Chemical Information Database for the Components of FgAG and WsAG

Besides the in-house Traditional Medicine Library in the UNIFI software, a systematic investigation of chemical components was conducted [34]. A self-built database of compounds that were isolated from FgAG and WsAG was established by searching online databases or internet search engines such as PubMed, Full-Text Database (CNKI), ChemSpider, Web of Science and Medline. Chemical information including the component name, molecular formula and structure of the components from the herbs were obtained from the database [56].

### 4.5. The Screening Analysis by the UNIFI Platform

To quickly identify the chemical compounds, the MS raw data, compressed with Waters Compression and Archival Tool v1.10, was automatically screened and identified by using the streamlined workflow of UNIFI 1.7.0 software (Waters, Manchester, UK) [30]. The parameters were as follows: the minimum peak area of 200 was set for 2D peak detection; the peak intensity of low energy over 1000 counts and the peak intensity of high energy over 200 counts were selected for 3D peak detection. Mass error up to ±5 ppm for identified compounds, retention time in the range of ±0.1 min was allowed to match the reference substance. The matching compounds would be generated predicted fragments from structure. The negative adducts containing +COOH and -H and positive adducts containing +H and +Na were selected in the analysis. Leucine-enkephalin was selected as the reference compound, and [M − H]^−^ 554.2620 was used for negative ion and [M + H]^+^ 556.2766 was used for positive ion [72].

### 4.6. The Metabolomics Analysis

The raw data were processed by MarkerLynx XS V4.1 software (Waters, Milford, CT, USA) for alignment, deconvolution, data reduction, etc. [73]. A MarkerLynx processing method was firstly created, and the main parameters were as follows: retention time range 0–26 min, mass range 100–1500 Da, mass tolerance 0.10, minimum intensity 5%, mass window 0.10, retention time window 0.20, marker intensity threshold 2000 counts and noise elimination level 6. After processing the data, the results could be showed in the Extended Statistics (XS) Viewer. *m*/*z*-RT pairs with corresponding intensities for all the detected peaks from each data file were listed. The same value of RT and *m*/*z* in different batched of samples were regarded as the same component. Then, multivariate statistical analysis was performed. Firstly, principal component analysis (PCA) was used to show the pattern recognition and maximum variation aiming to obtain the overview and classification. Secondly, orthogonal projections to latent structures discriminant analysis (OPLS-DA) in both positive and negative modes was performed in order to get the maximum separation between MsAG and FgAG group and to explore the potential chemical markers that contributed to the differences. Then, S-plots was created to provide visualization of the OPLS-DA predictive component loading to facilitate model interpretation. Meawhile, variable importance for the projection (VIP) was helpful to screen the different components, and the metabolites with VIP value (>3.0) were considered as potential markers [29]. In addition, the permutation test was performed to provide reference distributions of the R^2^/Q^2^-values that could indicate the statistical significance [30,31,32,33]. Simca 15.0 software (Umetrics, Malmö, Sweden) was used to show the analysis results [56,74].

## 5. Conclusions

In a comprehensive and unique phytochemical profile study, a total of 121 chemical compounds with different structural types were identified from WsAG and FgAG. The structural patterns included protopanaxdiol-type saponins, protopanaxtriol-type saponins, ocotillol-type saponins, oleanane-type saponins and other glyosides, organic acid and organic acid esters, steroids, etc. The results showed that WsAG and FgAG were rich in natural components. Furthermore, these 121 compounds were all shared constituents in them, meaning that they were similar in the kinds of compounds they contain. In metabolomic analysis, it was found that there indeed existed several differences in the contents of the constituents between FgAG and WsAG, and 22 robust known biomarkers enabling the differentiation were discovered. In a word, the results will fill the data gap in the study on the chemical constituents of WsAG and provide a reference for quantitative determinations in its quality control.

## Figures and Tables

**Table 1:** Compounds identified from FgAG and WsAG by UPLC-QTOF-MS^E^.

No.	t_R_ (min)	Formula	Calculated Mass (Da)	TheoreticalMass (Da)	Mass Error (ppm)	MS^E^ Fragmentation	Identification	Sources	Ref.
1	0.57	C_7_H_12_O_6_	192.0636	192.0634	1.0	191.0563[M − H]^−^, 173.0454[M − H-H_2_O]^−^, 127.0407[M − H-H_2_O-HCOOH]^−^, 109.0452[M − H-2H_2_O-HCOOH]^−^, 91.0352[M − H-3H_2_O-HCOOH]^−^	Quinic acid	WsAG, FgAG	s
2	0.64	C_12_H_22_O_11_	342.1162	342.1165	0.8	341.1092[M − H]^−^, 179.0562[M − H-Glu]^−^	*α*-Maltose	WsAG, FgAG	s
3	0.77	C_10_H_13_N_5_O_4_	267.0959	267.0968	−3.3	268.1031[M+H]^+^, 237.0874[M + H-CH_2_OH]^+^, 226.0898[M + H-CN_2_H_2_]^+^, 136.0612[M + H-Rib]^+^, 130.0495[M + H-CH_2_OH-C_4_N_4_H_3_]^+^	Adenosine	WsAG, FgAG	s
4	0.93	C_12_H_22_O_11_	342.1168	342.1162	1.7	341.1095[M − H]^−^, 287.1097[M − H-3H_2_O]^−^, 179.0563[M − H-Glu]^−^	Sucrose	WsAG, FgAG	s
5	0.95	C_9_H_11_NO_2_	165.0777	165.0782	−3.0	166.0850[M + H]^+^, 150.0589[M + H-NH_2_]^+^, 132.0486[M + H-H_2_O-NH_2_]^+^, 120.0807[M + H-HCOOH]^+^, 91.0559[M + H-CH-NH_2_-HCOOH]^+^	L-Phenylalanine	WsAG, FgAG	s
6 *	1.02	C_14_H_18_O_10_	346.0903	346.0900	1.0	345.0830[M − H]^−^, 327.0598[M − H-H_2_O]^−^, 309.0728[M − H-2H_2_O]^−^, 165.0195[M − H-Glu]^−^, 150.0115[M − H-Glu-CH_3_]^−^	Methyl gallate 3-*O*-*β*-d-glucoside	WsAG > FgAG VIP: 14.18 *p* < 0.001	s
7	1.51	C_11_H_12_N_2_O_2_	204.0899	204.0899	−0.1	203.0826[M − H]^−^, 141.0660[M − H-HCOOH-NH_2_]^−^, 129.0506[M − H-C_3_H_6_O_2_]^−^	L-Tryptophane	WsAG, FgAG	s
8	4.71	C_17_H_20_O_9_	368.1106	368.1107	−0.4	367.1033[M − H]^−^, 191.0754[M − H-C_10_H_9_O_3_]^−^, 193.0466[M − H-GluA]^−^, 177.0758[M − H-GluA-CH_3_]^−^, 127.0350[M − H-GluA-H_2_O-OCH_3_]^−^	3-*O*-trans-Feruloylquinic Acid	WsAG, FgAG	[36]
9	4.92	C_48_H_82_O_19_	962.5440	962.5450	−1.0	1007.5432[M + HCOO]^−^, 763.2898[M − H-2H_2_O-Glu]^−^, 815.4784[M − H-Rha]^−^, 781.3155[M − H-Glu]^−^, 635.2307[M − H-Glu-Rha-H_2_O]^−^, 437.1863[M − H-2Glu-Rha-3H_2_O]^−^	Majoroside F_6_	WsAG, FgAG	[37]
10	5.18	C_36_H_58_O_8_	618.4130	618.4132	−0.3	619.4203[M + H]^+^, 439.3712[M + H-Glu]^+^, 422.3451[M + H-Glu-OH]^+^, 383.2823[M + H-Glu-C_4_H_8_]^+^, 297.2336[M + H-Glu-C_9_H_16_O]^+^	Oleanolic acid -28-*O*-*β*-d-glucopyranoside	WsAG, FgAG	s
11	5.32	C_48_H_82_O_20_	978.5399	978.5397	−0.3	1023.5379[M + HCOO]^−^, 997.5331[M − H]^−^,815.4972[M − H-Glu]^−^, 797.4718[M − H-Glu-H_2_O]^−^, 653.3389[M − H-2Glu]^−^, 491.2724[M − H-3Glu]^−^	Yesanchinoside B	WsAG, FgAG	[38]
12 *	5.54	C_47_H_80_O_19_	948.5305	948.5294	1.2	993.5270[M + HCOO]^−^, 815.4921[M − H-Ara]^−^, 653.3392[M − H-2Glu-Ara]^−^, 473.3030[M − H-2Glu-Ara]^−^, 455.3922[M − H-2Glu-Ara-H_2_O]^−^, 391.2582[M − H-2Glu-Ara-C_6_H_12_O]^−^	Yesanchinoside C	WsAG > FgAG VIP: 6.18 *p* < 0.001	[38]
13 ^#^	5.78	C_48_H_82_O_19_	962.5443	962.5450	−1.0	1007.5436[M + HCOO]^−^, 961.5388[M − H]^−^, 799.4784[M − H-Glu]^−^, 637.2307[M − H-2Glu]^−^, 475.5863[M − H-3Glu]^−^	Notoginsenoside N	WsAG < FgAG VIP: 11.83 *p* < 0.001	[39]
14 ^#^	5.94	C_42_H_74_O_15_	818.5031	818.5028	0.4	863.5006[M + HCOO]^−^, 667.4323[M − H-Rha]^−^, 533.2329[M − H-C_6_H_13_O_2_-C_11_H_19_O]^−^, 506.3845[M − H-Glu-Rha]^−^, 477.2169[M − H-C_20_H_36_O_4_]^−^	Quinquenoside L_9_	WsAG < FgAG VIP: 7.20 *p* = 0.0002	s
15	5.94	C_42_H_72_O_14_	800.4915	800.4922	−0.9	801.4988[M + H]^+^, 621.4983[M + H-Glu-H_2_O]^+^, 459.3659[M + H-2Glu]^+^, 423.3450[M + H-2Glu-3H_2_O]^+^	Majoroside F_2_	WsAG, FgAG	[40]
16	6.25	C_26_H_34_O_11_	522.2097	522.2101	−0.7	567.2079[M + HCOO]^−^, 521.2204[M − H]^−^, 458.2935[M − H-H_2_O-C_2_H_5_O]^−^, 341.1396[M − H-Glu-H_2_O]^−^, 178.0559[M − H-C_20_H_23_O_5_]^−^	Urolignoside	WsAG, FgAG	[41]
17	6.48	C_54_H_92_O_23_	1108.6034	1108.6029	0.4	1153.6016[M + HCOO]^−^, 961.5452[M − H-Rha]^−^, 799.4902[M − H-Glu-Rha]^−^, 637.2950[M − H-2Glu-Rha]^−^, 475.2681[M − H-3Glu-Rha]^−^	Yesanchinoside E	WsAG, FgAG	[42]
18	6.77	C_47_H_80_O_18_	932.5348	932.5345	0.3	977.5318[M + HCOO]^−^, 931.5271[M − H]^−^, 799.4697[M − H-Ara]^−^, 769.4734[M − H-Glu]^−^, 637.3146[M − H-Glu-Ara]^−^	Quinquenoside F_6_	WsAG, FgAG	[37]
19	6.88	C_48_H_82_O_19_	962.5450	962.5450	−0.1	1007.5432[M + HCOO]^−^, 859.4881[M − H-C_5_H_10_O_2_]^−^, 799.4204[M − H-Glu]^−^, 696.4328[M − H-Glu-C_5_H_9_O]^−^, 637.3158[M − H-2Glu]^−^, 601.2316[M − H-2Glu-2H_2_O]^−^	Quinquenoside L_2_	WsAG, FgAG	[43]
20	7.01	C_42_H_72_O_14_	800.4914	800.4922	−1.0	845.4896[M + HCOO]^−^, 799.4836[M − H]^−^, 653.4319[M − H-Rha]^−^, 491.2475[M − H-Glu-Rha]^−^	(24*S*)-Pseudoginsenoside *F_11_*	WsAG, FgAG	s
21 *	7.05	C_47_H_80_O_18_	932.5349	932.5345	0.4	977.5346[M + HCOO]^−^, 840.4930[M − H-2H_2_O-C_4_H_7_]^−^, 799.4859[M − H-Xyl]^−^, 769.4735[M − H-Glu]^−^, 637.4321[M − H-Glu-Xyl]^−^	Notoginsenoside R_1_	WsAG > FgAG VIP: 24.59 *p* < 0.001	s
22 *	7.10	C_28_H_48_O	400.3723	400.3705	4.3	423.3620[M+Na]^+^, 382.2862[M + H-CH_3_]^+^, 339.2934[M + H-H_2_O-C_3_H_7_]^+^, 255. 2948[M + H-H_2_O-C_9_H_19_]^+^	Methylcholesta-7-en-3*β*-ol	WsAG > FgAG VIP: 4.69 *p* < 0.001	[44]
23	7.12	C_48_H_82_O_19_	962.5435	962.5450	−1.5	1007.54171[M + HCOO]^−^, 961.5329[M − H]^−^, 799.3722[M − H-Glu]^−^, 637.4321[M − H-2Glu]^−^, 475.3722[M − H-3Glu]^−^, 391.4833[M − H-3Glu-C_6_H_12_]^−^	Notoginsenoside R_6_	WsAG, FgAG	[42]
24	7.25	C_47_H_80_O_18_	932.5335	932.5345	−1.0	977.5317[M + HCOO]^−^, 931.5282[M − H]^−^, 799.4701[M − H-Xyl]^−^, 673.3294[M − H-Xyl-Glu]^−^, 475.2148[M − H-Xyl-2Glu]^−^	Notoginsenoside ST5	WsAG, FgAG	[45]
25	7.29	C_54_H_90_O_24_	1122.5818	1122.5822	0.4	1167.5812[M + HCOO]^−^, 1121.5747[M − H]^−^, 959.5120[M − H-Glu]^−^, 797.4669[M − H-2Glu]^−^, 473.4334[M − H-4Glu]^−^	Quinquenoside IV	WsAG, FgAG	[42]
26	7.40	C_42_H_72_O_14_	800.4918	800.4922	−0.5	845.4900[M + HCOO]^−^, 784.4683[M − H-CH_3_]^−^, 637.4340[M − H-Glu]^−^, 471.3787[M − H-2Glu]^−^	Ginsenoside Rg_1_	WsAG, FgAG	s
27	7.47	C_48_H_82_O_18_	946.5491	946.5501	−1.0	991.5473[M + HCOO]^−^, 945.5413[M − H]^−^, 783.5142[M − H-Glu]^−^, 637.4125[M − H-Glu-Rha]^−^, 475.5147[M − H-2Glu-Rha]^−^	Ginsenoside Re	WsAG, FgAG	s
28	7.47	C_28_H_48_O	400.3715	400.3705	2.4	423.3617[M+Na]^+^, 401.3540[M + H]^+^, 383.2861[M + H-H_2_O]^+^, 325.2982[M + H-H_2_O-CH_3_-C_3_H_7_]^+^, 284.1420[M + H-H_2_O-C_7_H_15_]^+^, 175.1221[M + H-H_2_O-C_15_H_28_]^+^	Campesterol	WsAG, FgAG	a
29	7.69	C_45_H_74_O_17_	886.4926	886.4925	−0.1	885.4853[M − H]^−^, 799.4748[M − H-Mal]^−^, 637.4303[M − H-Mal-Glu]^−^, 475.3751[M − H-Mal-2Glu]^−^	Malonyl-ginsenoside Rg_1_	WsAG, FgAG	[46]
30	7.85	C_42_H_72_O_14_	800.4929	800.4922	0.8	845.4911[M + HCOO]^−^, 799.4837[M − H]^−^, 653.3687[M − H-Rha]^−^, 491.2354[M − H-Rha-Glu]^−^	Quinquenoside L_11_	WsAG, FgAG	s
31 ^#^	7.84	C_51_H_84_O_21_	1032.5505	1032.5532	2.6	1031.5414[M − H]^−^, 945.5212[M − H-Mal]^−^, 783.4173[M − H-Mal-Glu]^−^, 637.4385[M − H-Mal-Rha-Glu-Ac]^−^, 475.3932[M − H-Mal-2Glu-Rha]^−^	Malonyl-ginsenoside Re	WsAG < FgAG VIP: 5.65 *p* = 0.0060	[39]
32	7.94	C_47_H_80_O_19_	948.5282	948.5294	−1.2	947.5209[M − H]^−^, 815.4786[M − H-Xyl]^−^, 653.2758[M − H-Glu-Xyl]^−^, 491.1787[M − H-2Glu-Xyl]^−^	Vinaginsenoside R_6_	WsAG, FgAG	[39]
33	8.15	C_47_H_80_O_17_	916.5409	916.5396	1.4	961.5377[M + HCOO]^−^, 915.5306[M − H]^−^, 783.4819[M − H-Ara]^−^, 753.4732[M − H-Glu]^−^, 621.4290[M − H-Ara-Glu]^−^, 459.4687[M − H-2Glu-Ara]^−^	Quinquenoside L_14_	WsAG, FgAG	[47]
34	8.23	C_44_H_74_O_15_	842.4996	842.5028	−3.6	887.4978[M + HCOO]^−^, 841.4939[M − H]^−^, 799.4833[M − H-Ac]^−^, 695.4459[M − H-Glu]^−^, 653.4321[M − H-Ac-Rha]^−^, 684.3932[M − H-CH_3_-C_8_H_14_O_2_]^−^, 491.4219[M − H-Rha-Glu-Ac]^−^	Vinaginsenoside R_1_	WsAG, FgAG	[48]
35 *	8.26	C_54_H_94_O_24_	1126.6162	1126.6135	2.4	1171.6110[M + HCOO]^−^, 1125.6094[M − H]^−^, 975.5349 [M − H-Xyl-H_2_O]^−^, 963.5502[M − H-Glu]^−^, 801.3547[M − H-2Glu]^−^, 831.3214[M − H-Glu-Xyl]^−^, 507.3214[M − H-3Glu-Xyl]^−^	Quinquenoside F_3_	WsAG > FgAG VIP: 3.35 *p* < 0.001	s
36	8.57	C_44_H_74_O_15_	842.5028	842.5012	−1.7	887.4995[M + HCOO]^−^, 841.4939[M − H]^−^, 637.4321[M − H-Glu-Ac]^−^, 475.3030[M − H-2Glu-Ac]^−^, 391.4158[M − H-2Glu-Ac-C_6_H_11_]^−^	Acetyl-Ginsenoside Rg_1_	WsAG, FgAG	[39]
37 *	8.59	C_41_H_70_O_13_	770.4808	770.4816	−1.0	815.4798[M + HCOO]^−^, 769.4722[M − H]^−^, 637.4321[M − H-Ara]^−^, 475.2678[M − H-Ara-Glu]^−^, 391.1748[M − H-Ara-Glu-C_6_H_11_]^−^	Notoginsenoside R_2_	WsAG > FgAG VIP: 4.83 *p* < 0.001	[42]
38	8.63	C_48_H_82_O_19_	962.5423	962.5450	−2.7	1007.5405[M + HCOO]^−^, 961.5371[M − H]^−^, 815.4317[M − H-Rha]^−^, 799.4622[M − H-Glu]^−^, 653.4385[M − H-Glu-Rha]^−^, 617.4316[M − H-Glu-Rha-H_2_O]^−^, 491.2912[M − H-2Glu-Rha]^−^	Majoroside F_5_	WsAG, FgAG	[37]
39	8.64	C_30_H_48_O_2_	440.3646	440.3654	−1.9	441.3719[M + H]^+^, 423.3606[M + H-H_2_O]^+^, 339.2908[M + H-HCOOH-C_4_H_8_]^+^, 248.2948[M + H-C_14_H_24_]^+^, 203.1849[M + H-HCOOH-C_14_H_24_]^+^	Deoxyoleanolic acid	WsAG, FgAG	[46]
40	8.78	C_48_H_80_O_18_	944.5338	944.5345	−0.6	989.5320[M + HCOO]^−^, 943.5250[M − H]^−^, 781.4541[M − H-Glu]^−^, 619.4143[M − H-2Glu]^−^, 457.5876[M − H-3Glu]^−^	Quinquenoside L_1_	WsAG, FgAG	[49]
41	8.83	C_53_H_88_O_23_	1092.5718	1092.5716	0.2	1137.5696[M + HCOO]^−^, 1091.5641[M − H]^−^, 959.5571[M − H-Xyl]^−^, 929.4601[M − H-Glu]^−^, 797.4852[M − H-Glu-Xyl]^−^	Yesanchinoside G	WsAG, FgAG	[50]
42 *	8.89	C_47_H_78_O_17_	914.5225	914.5239	−1.5	959.5225[M + HCOO]^−^, 913.5147[M − H]^−^, 733.2547[M − H-H_2_O-Glu]^−^, 619.4527[M − H-Glu-Xyl]^−^, 457.3254[M − H-2Glu-Xyl]^−^	Quinquenoside L_8_	WsAG > FgAG VIP: 3.97 *p* = 0.0004	s
43	8.97	C_48_H_82_O_19_	962.5438	962.5450	−1.2	1007.5420[M + HCOO]^−^, 946.5212[M − H-CH_3_]^−^, 781.4533[M − H-Glu-H_2_O]^−^, 637.4321[M − H-2Glu]^−^, 475.3932[M − H-3Glu]^−^	Majoroside F_1_	WsAG, FgAG	[40]
44 *	9.14	C_42_H_70_O_13_	782.4325	782.4816	1.2	781.4747[M − H]^−^, 619.4181[M − H-Glu]^−^, 457.4798[M − H-2Glu]^−^, 376.4797[M − H-2Glu-C_6_H_9_]^−^	Quinquenoside F_1_	WsAG > FgAG VIP: 4.10 *p* = 0.0004	[51]
45	9.27	C_15_H_10_O_6_	286.0480	286.0477	0.8	285.0407[M − H]^−^, 227.0521[M − H-C_2_H_2_O_2_]^−^, 151.0037[M − H-C_8_H_6_O_2_]^−^, 106.0148[M − H-C_9_H_7_O_4_]^−^, 112.0351[M − H-C_9_H_5_O_5_]^−^	Kaempferol	WsAG, FgAG	s
46 ^#^	9.32	C_53_H_86_O_24_	1118.5514	1118.5509	0.5	1117.5436[M − H]^−^, 1040.5481[M − H-CH_3_OH]^−^, 955.3219[M − H-Glu]^−^, 793.2905[M − H-2Glu]^−^, 453.1095[M − H-3Glu-GluA]^−^	Ginsenoside R_OA_	WsAG < FgAG VIP: 12.60 *p* < 0.001	[52]
47 *	9.54	C_41_H_70_O_14_	786.4775	786.4766	1.1	831.4775[M + HCOO]^−^, 767.4297[M − H-H_2_O]^−^, 653.4318[M − H-Xyl]^−^, 491.2015[M − H-Glu-Xyl]^−^	Majonoside R_2_	WsAG > FgAG VIP: 25.80 *p* < 0.001	[39]
48	9.61	C_48_H_80_O_19_	960.5285	960.5294	−0.9	1005.5267[M + HCOO]^−^, 941.5316[M − H-H_2_O]^−^, 797.4287[M − H-Glu]^−^, 635.3221[M − H-2Glu]^−^, 473.2684[M − H-3Glu]^−^	Notoginsenoside G	WsAG, FgAG	[42]
49	9.68	C_42_H_72_O_14_	800.4922	800.4922	0.0	845.4904[M + HCOO]^−^, 799.4844[M − H]^−^, 783.2451[M − H-Rha]^−^, 621.3547[M − H-Glu-Rha]^−^	Pseudo-ginsenoside F_11_	WsAG, FgAG	s
50	9.73	C_36_H_62_O_10_	654.4340	654.4343	−0.4	699.4322[M + HCOO]^−^, 653.4262[M − H]^−^, 635.4312[M − H-H_2_O]^−^, 491.3254[M − H-Glu]^−^	Pseudo-ginsenoside RT_5_	WsAG, FgAG	s
51	9.78	C_36_H_62_O_10_	654.4337	654.4343	−0.9	655.4410[M + H]^+^, 599.4418[M − H-3H_2_O]^+^, 493.3437[M − H-Glu]^+^, 457.2651[M − H-Glu-2H_2_O]^+^	Pseudo-ginsenoside RT_4_	WsAG, FgAG	[39]
52	9.82	C_59_H_100_O_27_	1240.6458	1240.6452	0.5	1239.6380[M − H]^−^, 1107.6376[M − H-Xyl]^−^, 954.6930[M − H-Xyl-Glu]^−^, 783.4833[M − H-Xyl-2Glu]^−^, 621.4431[M − H-Xyl-3Glu]^−^, 459.4943[M − H-Xyl-4Glu]^−^	Ginsenoside Ra_3_	WsAG, FgAG	[53]
53	9.96	C_41_H_70_O_13_	770.4810	770.4816	−0.8	815.4804[M + HCOO]^−^, 751.4804[M − H-H_2_O]^−^, 637.4904[M − H-Ara]^−^, 475.3804[M − H-Glu-Ara]^−^	Ginsenoside F_5_	WsAG, FgAG	[39]
54	9.98	C_58_H_98_O_26_	1210.6350	1210.6346	0.3	1209.6272[M − H]^−^, 1077.5814[M − H-Xyl]^−^, 945.4706[M − H-Xyl-Ara]^−^, 783.4803[M − H-Xyl-Ara-Glu]^−^, 459.4799[M − H-Xyl-Ara-3Glu]^−^	Ginsenoside Ra_2_	WsAG, FgAG	[53]
55	10.02	C_41_H_66_O_11_	734.4590	734.4605	−2.1	735.4663[M + H]^+^, 589.3646[M + H-Rha]^+^, 457.3705[M + H-Rha-Ara]^+^, 441.5712[M + H-Rha-Ara-HCOOH]^+^	Eleutheroside K	WsAG, FgAG	[54]
56	10.04	C_48_H_80_O_18_	944.5320	944.5345	−2.6	989.5302[M + HCOO]^−^, 943.5263[M − H]^−^, 781.4839[M − H-Glu]^−^, 619.4206[M − H-2Glu]^−^, 457.5701[M − H-3Glu]^−^	Quinquenoside L_6_	WsAG, FgAG	-
57	10.07	C_30_H_48_O_2_	440.3638	440.3654	−3.6	441.3717[M + H]^+^, 394.3508[M + H-H_2_O-CHO]^+^, 328.3504[M + H-CHO-C_6_H_12_]^+^, 219.1792[M + H-C_15_H_26_O]^+^, 205.1619[M + H-H_2_O-C_15_H_22_O]^+^	3*β*-Hydroxyolean-12-en-28-al	WsAG, FgAG	a
58	10.14	C_59_H_100_O_27_	1240.6452	1240.6462	0.8	1285.6444[M + HCOO]^−^, 1107.5976[M − H-Xyl]^−^, 945.4900[M − H-Xyl-Glu]^−^, 783.4835[M − H-Xyl-2Glu]^−^, 459.4929[M − H-Xyl-4Glu]^−^	Notoginsenoside Fa	WsAG, FgAG	[53]
59	10.24	C_54_H_92_O_23_	1108.6039	1108.6029	0.8	1153.6021[M + HCOO]^−^, 1107.5961[M − H]^−^, 943.5414[M − H-Glu]^−^, 763.4784[M − H-2Glu]^−^, 615.4417[M − H-3Glu]^−^	Ginsenoside Rb_1_	WsAG, FgAG	s
60	10.24	C_30_H_48_O	424.3692	424.3705	−3.1	425.3765[M + H]^+^, 409.3102[M + H-H_2_O]^+^, 371.3759[M + H-CH_3_-C_3_H_5_]^+^, 189.1614[M + H-C_16_H_26_O]^+^, 205.1775[M + H-C_15_H_26_O]^+^	Olean-18-en-3-one	WsAG, FgAG	[55]
61	10.31	C_57_H_94_O_26_	1194.6054	1194.6033	1.7	1193.5981[M − H]^−^, 1077.5402[M − H-mal]^−^, 945.5097[M − H-mal-Glu]^−^, 783.4906[M − H-mal-2Glu]^−^, 621.4906[M − H-mal-3Glu]^−^	Malonyl-ginsenoside Rb_1_	WsAG, FgAG	[53]
62	10.33	C_42_H_72_O_13_	784.4975	784.4973	0.3	829.4957[M + HCOO]^−^, 768.4744[M − H-CH_3_]^−^, 635.4330[M − H-Rha]^−^, 471.3782[M − H-Glu-Rha]^−^	20(*R*)-Ginsenoside Rg_2_	WsAG, FgAG	s
63	10.35	C_36_H_62_O_9_	638.4391	638.4394	−0.4	683.4373[M + HCOO]^−^, 637.4313[M − H]^−^, 475.2658[M − H-Glu]^−^, 457.2235[M − H-Glu-H_2_O]^−^	20(*S*)-Ginsenoside Rh_1_	WsAG, FgAG	s
64	10.36	C_41_H_70_O_13_	770.4817	770.4816	0.0	815.4799[M + HCOO]^−^, 678.4450[M − H-2H_2_O-C_4_H_7_]^−^, 637.4321[M − H-Ara]^−^, 590.2706[M − H-C_4_H_7_-C_9_H_16_]^−^, 475.2622[M − H-Glu-Ara]^−^	Ginsenoside F_3_	WsAG, FgAG	[39]
65	10.39	C_53_H_90_O_22_	1078.5931	1078.5924	0.6	1123.5913[M + HCOO]^−^, 943.5423[M − H-Araf]^−^, 854.4890[M − H-H_2_O-Araf-C_4_H_7_]^−^, 763.4850[M − H-Glu-Araf]^−^	Ginsenoside Rc	WsAG, FgAG	s
66	10.42	C_36_H_60_O_8_	620.4276	620.4288	−1.9	621.4349[M + H]^+^, 603.4238[M + H-H_2_O]^+^, 441.3714[M + H-Glu]^+^, 423.3612[M + H-Glu-H_2_O]^+^, 350.2971[M + H-Glu-2H_2_O-C_4_H_7_]^+^, 341.1160[M + H-Glu-C_6_H_12_O]^+^	Ginsenoside Rh_4_	WsAG, FgAG	[56]
67	10.46	C_58_H_98_O_26_	1210.6353	1210.6346	0.6	1209.6275[M − H]^−^, 1077.5914[M − H-Xyl]^−^, 945.4807[M − H-Xyl-Ara]^−^, 783.4687[M − H-Xyl-Ara-Glu]^−^, 459.4329[M − H-Xyl-Ara-3Glu]^−^	Ginsenoside Ra_1_	WsAG, FgAG	[53]
68	10.58	C_56_H_92_O_25_	1164.5932	1164.5928	0.3	1163.5859[M − H]^−^, 1119.5976[M − H-CO_2_]^−^, 1077.6021[M − H-Mal]^−^, 1031.5694[M − H-Araf]^−^, 945.4900[M − H-Araf-Mal]^−^, 783.4835[M − H-Glu-Araf-Mal]^−^	Malonyl-ginsenoside Rc	WsAG, FgAG	[53]
69	10.62	C_48_H_76_O_19_	956.4976	956.4981	−0.5	955.4903[M − H]^−^, 783.4214[M − H-GluA]^−^, 631.4157[M − H-2Glu]^−^, 459.4174[M − H-2Glu-GluA]^−^	Ginsenoside Ro	WsAG, FgAG	s
70 ^#^	10.69	C_30_H_46_O_2_	438.3486	438.3498	−2.7	439.3563[M + H]^+^, 424.3600[M + H-CH_3_]^+^, 411.1114[M + H-CO]^+^, 233.1676[M + H-C_15_H_24_]^+^, 205.1928[M + H-C_15_H_22_O_2_]^+^, 190.1778[M + H-C_16_H_23_O_2_]^+^	3,11-dioxo-*β*-amyrene	WsAG < FgAG VIP: 6.49 *p* < 0.001	[57]
71	10.70	C_53_H_84_O_23_	1088.5402	1088.5403	−0.1	1087.5326[M − H]^−^, 955.5235[M − H-Ara]^−^, 925.4610[M − H-Glu]^−^, 793.2350[M − H-Ara-Glu]^−^, 455.4611[M − H-Ara-2Glu-GluA]^−^	Stipuleanoside R2	WsAG, FgAG	[39]
72	10.78	C_53_H_90_O_22_	1078.5924	1078.5924	0.0	1123.5906[M + HCOO]^−^, 913.5403[M − H-Glu]^−^, 779.4886[M − H-Glu-Ara]^−^, 615.4431[M − H-2Glu-Ara]^−^	Ginsenoside Rb_2_	WsAG, FgAG	s
73	10.79	C_53_H_90_O_22_	1078.5924	1078.5924	0.0	1123.5320[M + HCOO]^−^, 913.4581[M − H-Glu]^−^, 779.3696[M − H-Glu-Xyl]^−^, 615.4912[M − H-2Glu-Xyl]^−^, 451.3672[M − H-3Glu-Xyl]^−^	Ginsenoside Rb_3_	WsAG, FgAG	s
74	10.89	C_55_H_92_O_23_	1120.6009	1120.6029	−1.8	1165.5991[M + HCOO]^−^, 1077.3151[M − H-Xyl]^−^, 945.5076[M − H-Ara-Xyl]^−^, 783.3942[M − H-Ara-Xyl-Glu]^−^, 621.4742[M − H-Ara-Xyl-2Glu]^−^	Notoginsenoside Fc	WsAG, FgAG	[53]
75	10.94	C_56_H_92_O_25_	1164.5937	1164.5928	0.8	1163.5864[M − H]^−^, 1077.5570[M − H-Mal]^−^, 945.5302[M − H-Ara-Mal]^−^, 783.4540[M − H-Glu-Ara-Mal]^−^, 621.4570[M − H-2Glu-Ara-Mal]^−^	Malonyl-ginsenoside Rb_2_	WsAG, FgAG	[53]
76	11.01	C_56_H_94_O_24_	1150.6138	1150.6135	0.3	1195.6120[M + HCOO]^−^, 1149.6060[M − H]^−^, 1107.4997[M − H-Ac]^−^, 987.4976[M − H-Glu]^−^, 945.6047[M − H-Glu-Ac]^−^, 783.4864[M − H-2Glu-Ac]^−^	Quinquenoside R_1_	WsAG, FgAG	[53]
77	11.02	C_47_H_74_O_18_	926.4864	926.4875	−1.2	925.4791[M − H]^−^, 793.4272[M − H-Ara]^−^, 612.3784[M − H-GluA-Ara]^−^, 540.3784[M − H-Glu-C_14_H_21_O]^−^, 455.2841[M − H-Glu-Ara-GluA]^−^	Chikusetsu saponin IV	WsAG, FgAG	[39]
78	11.14	C_56_H_92_O_25_	1164.4967	1164.4958	0.8	1163.4925[M − H]^−^, 1077.5760[M − H-Mal]^−^, 945.5503[M − H-Mal-Xyl]^−^, 783.4735[M − H-Mal-Xyl-Glu]^−^, 621.4269[M − H-Mal-Xyl-2Glu]^−^	Malonyl-ginsenoside Rb_3_	WsAG, FgAG	[53]
79	11.16	C_36_H_62_O_9_	638.4395	638.4394	0.2	683.4366[M + HCOO]^−^, 637.4317[M − H]^−^, 475.2574[M − H-Glu]^−^, 457.2147[M − H-Glu-H_2_O]^−^	20(*R*)-ginsenoside Rh_1_	WsAG, FgAG	s
80	11.18	C_43_H_72_O_15_	828.4864	828.4871	−0.8	873.4846[M + HCOO]^−^, 784.4798[M − H-COCH_3_]^−^, 695.2912[M − H-Xyl]^−^, 491.4938[M − H-Xyl-Glu-Ac]^−^, 455.2535[M − H-Xyl-Glu-Ac-2H_2_O]^−^	Vinaginsenoside R_2_	WsAG, FgAG	[39]
81 ^#^	11.20	C_48_H_82_O_17_	930.5546	930.5552	−0.7	929.5474[M − H]^−^, 767.4642[M − H-Glu]^−^, 605.4365[M − H-2Glu]^−^, 443.1196[M − H-3Glu]^−^	Vinaginsenosides R_3_	WsAG < FgAG VIP: 7.60 *p* < 0.001	[58,59]
82	11.34	C_42_H_66_O_14_	794.4447	794.4453	−0.8	793.4368[M − H]^−^, 631.3279[M − H-Glu]^−^, 613.4222[M − H-Glu-H_2_O]^−^, 569.2927[M − H-Glu-HCOOH]^−^, 455.1562[M − H-Glu-GluA]^−^	Chikusetsu saponin II	WsAG, FgAG	[39]
83	11.36	C_48_H_82_O_18_	946.5508	946.5501	0.7	991.5490[M + HCOO]^−^, 945.5430[M − H]^−^, 783.5147[M − H-Glu]^−^, 459.3241[M − H-3Glu]^−^	Ginsenoside Rd	WsAG, FgAG	s
84	11.39	C_55_H_92_O_23_	1120.6029	1120.6049	1.7	1119.5941[M − H]^−^, 1077.5699[M − H-Ac]^−^, 943.4874[M − H-Ac-Ara]^−^, 779.1224[M − H-Ac-Ara-Glu]^−^, 451.2649[M − H-Ac-Ara-3Glu]^−^	Ginsenoside Rs_1_	WsAG, FgAG	s
85	11.52	C_51_H_84_O_21_	1032.5503	1032.5505	−0.2	1031.5425[M − H]^−^, 987.5520[M − H-CO_2_]^−^, 945.5192[M − H-mal]^−^, 783.3540[M − H-mal-Glu]^−^, 621.2570[M − H-mal-2Glu]^−^, 459.3458[M − H-mal-3Glu]^−^	Malonyl-ginsenoside Rd	WsAG, FgAG	[53]
86	11.70	C_55_H_92_O_23_	1120.6039	1120.6029	0.8	1165.6021[M + HCOO]^−^, 1119.5961[M − H]^−^, 987.3684[M − H-Ara]^−^, 914.4587[M − H-Glu-Ac]^−^, 458.5471[M − H-3Glu-Ara-Ac]^−^	Ginsenoside Rs_2_	WsAG, FgAG	s
87	11.88	C_48_H_82_O_18_	946.5501	946.5500	−0.1	991.5482[M + HCOO]^−^, 783.4871[M − H-Glu]^−^, 603.4416[M − H-2Glu]^−^	Gypenoside XVII	WsAG, FgAG	s
88	12.20	C_19_H_36_O_5_	344.2565	344.2563	0.6	343.2486[M − H]^−^, 329.0232[M − H-CH_3_]^−^, 311.2112[M − H-H_2_O-CH_3_]^−^, 294.1609[M − H-H_2_O-OCH_3_]^−^, 255.1494[M − H-OCH_3_-C_4_H_9_]^−^, 242.1610[M − H-OCH_3_-C_5_H_11_]^−^, 228.2523[M − H-OCH_3_-C_6_H_12_]^−^	Methyl-9,10,11-trihydroxy-12- octadecenoate	WsAG, FgAG	[60]
89	12.27	C_47_H_80_O_17_	916.5398	916.5396	0.2	961.5380[M + HCOO]^−^, 866.4901[M − H-H_2_O-CH_2_OH]^−^, 783.4749[M − H-Ara]^−^, 753.4664[M − H-Glu]^−^, 621.4616[M − H-Glu-Ara]^−^, 459.4008[M − H-2Glu-Ara]^−^	Notoginsenoside Fe	WsAG, FgAG	[39]
90	12.38	C_50_H_84_O_19_	988.5594	988.5607	−1.3	1033.5576[M + HCOO]^−^, 987.5539[M − H]^−^, 945.5428[M − H-COCH_3_]^−^, 809.4326[M − H-2H_2_O-C_8_H_14_O_2_]^−^, 797.4813[M − H-Glu-C_2_H_4_]^−^	Quinquenoside III	WsAG, FgAG	[61]
91 ^#^	12.48	C_47_H_80_O_17_	916.5385	916.5396	−1.1	961.5385[M + HCOO]^−^, 900.5146[M − H-CH_3_]^−^, 783.49.6[M − H-Ara]^−^, 630.4290[M − H-Glu-2H_2_O-C_5_H_9_]^−^, 621.3500[M − H-Glu-Ara]^−^, 459.2328[M − H-2Glu-Ara]^−^	Chikusetsu saponin III	WsAG < FgAG VIP: 12.78 *p* < 0.001	[39]
92	12.57	C_30_H_46_O_2_	766.4855	766.4867	−1.6	767.4928[M + H]^+^, 749.3674[M + H-H_2_O]^+^, 621.2398[M + H-Rha]^+^, 459.2280[M + H-Glu-Rha]^+^, 207.1780[M + H-Glu-Rha-C_16_H_26_O]^+^	(20*E*)-Ginsenoside F_4_	WsAG, FgAG	[62]
93	12.68	C_47_H_80_O_17_	916.5399	916.5396	0.3	961.5381[M + HCOO]^−^, 814.4616[M − H-H_2_O-C_6_H_11_]^−^, 783.4923[M − H-Xyl]^−^, 621.4390[M − H-Glu-Xyl]^−^	Gypenoside IX	WsAG, FgAG	[53]
94	13.14	C_42_H_70_O_14_	798.4748	798.4765	−2.1	797.4676[M − H]^−^, 651.4246[M − H-Rha]^−^, 489.4158[M − H-Glu-Rha]^−^	Ginsenoside Rg_8_	WsAG, FgAG	[63]
95	13.18	C_52_H_86_O_19_	1014.5756	1014.5763	−0.6	1059.5738[M + HCOO]^−^, 1013.5685[M − H]^−^, 945.5933[M − H-C_4_H_5_O]^−^, 851.4510[M − H-Glu]^−^, 833.4903[M − H-Glu-H_2_O]^−^, 620.2875[M − H-2Glu-C_4_H_5_O]^−^, 458.2663[M − H-3Glu-C_4_H_5_O]^−^	Quinquenoside I	WsAG, FgAG	[61]
96	13.31	C_48_H_82_O_17_	930.5548	930.5552	−0.4	929.5470[M − H]^−^, 783.4642[M − H-Rha]^−^, 767.4695[M − H-Glu]^−^, 621.4365[M − H-Glu-Rha]^−^, 459.3956[M − H-2Glu-Rha]^−^	Gypenoside X	WsAG, FgAG	-
97	13.43	C_42_H_70_O_12_	766.4861	766.4867	−0.8	765.4783[M + HCOO]^−^, 610.2361[M − H-CH_2_OH-C_8_H_13_-CH_3_]^−^, 603.2375[M − H-Glu]^−^, 441.1811[M − H-2Glu]^−^, 340.2323[M − H-C_30_H_49_O]^−^	Ginsenoside Rk_1_	WsAG, FgAG	[39]
98	13.50	C_47_H_74_O_18_	926.4862	926.4875	−1.4	925.4789[M − H]^−^, 793.4879[M − H-Ara]^−^, 731.4457 [M − H-Ara-HCOOH]^−^, 727.4338[M − H-Glu-H_2_O]^−^, 659.4254[M − H-Glu-C_3_H_4_-HCOOH]^−^, 569.4945 [M − H-Ara-Glu-HCOOH]^−^, 455. 4979 [M − H-Ara- Glu-GluA]^−^	Chikusetsu saponin Ib	WsAG, FgAG	[39]
99 ^#^	13.52	C_42_H_72_O_13_	784.4974	784.4973	−0.1	783.4896[M − H]^−^, 737.4755[M − H-CH_2_OH-CH_3_]^−^, 660.4330[M − H-3H_2_O-C_5_H_9_]^−^, 621.4361[M − H-Glu]^−^, 459.3782[M − H-2Glu]^−^	Ginsenoside F_2_	WsAG < FgAG VIP: 17.01 *p* < 0.001	[39]
100	13.57	C_18_H_30_O_4_	310.2141	310.2144	−1.0	309.2068[M − H]^−^, 291.1960[M − H-H_2_O]^−^, 185.1181[M − H-COOH-C_6_H_9_]^−^, 171.1024[M − H-CH_2_COOH-C_6_H_9_]^−^	13S-hydroperoxy-9Z,11E,15Z- octadecatrienoic acid	WsAG, FgAG	[64]
101	13.62	C_36_H_60_O_7_	604.4334	604.4339	−0.8	605.4407[M + H]^+^, 586.4285[M + H-H_2_O]^+^, 443.3860[M + H-Glu]^+^, 405.3657[M + H-Glu-H_2_O]^+^, 333.0939[M + H-2H_2_O-C_16_H_26_O]^+^, 296.1006[M + H-Glu-H_2_O-C_8_H_13_]^+^	Isoginsenoside Rh_3_	WsAG, FgAG	[65]
102 *	13.93	C_42_H_66_O_14_	794.4449	794.4453	−0.5	793.4387[M − H]^−^, 613.3751[M − H-Glu]^−^, 569.3830[M − H-Glu-H_2_O-CO_2_]^−^	Chikusetsusaponin Iva	WsAG > FgAG VIP:16.17 *p* < 0.001	[53]
103	14.51	C_42_H_72_O_13_	784.4967	784.4973	−0.7	829.4951[M + HCOO]^−^, 783.4892[M − H]^−^, 621.4442[M − H-Glu]^−^, 459.3684[M − H-2Glu]^−^	20(*R*)-Ginsenoside Rg_3_	WsAG, FgAG	s
104	14.64	C_17_H_30_O_2_	266.2246	266.2246	−0.1	311.2228[M + HCOO]^−^, 168.1023[M − H-C_7_H_13_]^−^, 154.1074[M − H-C_8_H_15_]^−^, 137.2250[M − H-C_7_H_13_-OCH_3_]^−^, 115.0352[M − H-C_4_H_7_-C_6_H_9_O]^−^, 96.0352[M − H-C_11_H_21_O]^−^	5-Hexenoic acid, 10-undecenyl ester	WsAG, FgAG	a
105 ^#^	14.74	C_18_H_28_O_2_	276.2081	276.2089	−2.9	277.2156[M + H]^+^, 150.1312[M + H-C_7_H_11_O_2_]^+^, 137.0951[M + H-H_2_O-C_9_H_14_]^+^, 110.1017[M + H-C_10_H_15_O_2_]^+^	Palmitoleic acid	WsAG < FgAG VIP: 8.57 *p* < 0.001	s
106	14.75	C_42_H_72_O_13_	784.4940	784.4973	−4.1	829.4951[M + HCOO]^−^, 783.4865[M − H]^−^, 621.4942[M − H-Glu]^−^, 459.4578[M − H-2Glu]^−^, 441.5214[M − H-2Glu-H_2_O]^−^	20(*S*)-Ginsenoside Rg_3_	WsAG, FgAG	s
107	14.90	C_41_H_70_O_12_	754.4874	754.4867	0.8	799.4856[M + HCOO]^−^, 621.3141[M − H-Xyl]^−^, 459.3887[M − H-Glu-Xyl]^−^, 351.2556[M − H-Xyl-H_2_O-C_16_H_28_O_2_]^−^, 275.1442[M − H-Glu-Xyl-2C_6_H_11_]^−^	Gypenoside XIII	WsAG, FgAG	[66]
108	14.98	C_41_H_64_O_13_	764.4345	764.4347	−0.3	763.4272[M − H]^−^, 613.3766[M − H-Xyl]^−^, 569.3856[M − H-Xyl-HCOOH]^−^	Pseudo-ginsenoside Rp_1_	WsAG, FgAG	[39]
109	15.05	C_17_H_30_O_2_	266.2244	266.2246	−0.7	311.2226[M + HCOO]^−^, 222.1128[M − H-C_3_H_7_]^−^, 139.0826[M − H-C_9_H_19_]^−^, 127.1127[M − H-C_8_H_11_O_2_]^−^	(2E,4E)-Hydroprene	WsAG, FgAG	a
110	15.75	C_43_H_68_O_14_	808.4610	808.4609	0.1	807.4532[M − H]^−^, 609.3820[M − H-Glu-H_2_O]^−^, 455.3519[M − H-Glu-Glu acid methyl ester]^−^, 319.1792[M − H-Glu acid methyl ester-C_21_H_32_]^−^	Chikusetsusaponin IVa methyl ester	WsAG, FgAG	[39]
111	15.98	C_18_H_30_O_3_	294.2194	294.2195	−0.3	293.2122[M − H]^−^, 275.2013[M − H-H_2_O]^−^, 171.1024[M − H-C_9_H_15_]^−^, 121.1020[M − H-C_9_H_15_O_3_]^−^	(*E*,*E*)-9-Oxooctadeca-10,12-dienoic acid	WsAG, FgAG	[34]
112 *	16.90	C_36_H_62_O_8_	622.4431	622.4445	−2.1	667.4442[M + HCOO]^−^, 621.4360[M − H]^−^, 459.2656[M − H-Glu]^−^, 441.4772[M − H-Glu-H_2_O]^−^	Ginsenoside Rh_2_	WsAG > FgAG VIP: 4.68 *p* = 0.0032	s
113	17.34	C_18_H_32_O_3_	296.2347	296.2351	1.4	295.2274[M − H]^−^, 278.2172[M − H-H_2_O]^−^, 233.2273[M − H-HCOOH-O]^−^, 184.1182[M − H-C_8_H_15_]^−^, 171.1023[M − H-C_9_H_16_]^−^, 148.1125[M − H-C_8_H_15_O_2_]^−^, 125.1174[M − H-H_2_O-C_10_H_17_O]^−^	9-Hydroxyoctadeca-10,12-dienoic acid	WsAG, FgAG	[67]
114	17.37	C_42_H_70_O_12_	766.4860	766.4867	−0.9	811.4933[M + HCOO]^−^, 747.4834[M − H-H_2_O]^−^, 603.4833[M − H-Glu]^−^, 585.4309[M − H-Glu]^−^, 459.0768[M − H-Glu-Rha]^−^, 421.4457[M − H-Glu-Rha]^−^	Ginsenoside Rg_5_	WsAG, FgAG	[53]
115 ^#^	17.45	C_18_H_30_O_2_	278.2245	278.2246	−0.1	279.2321[M + H]^+^, 218.1936[M + H-HCOOH-CH_3_]^+^, 184.1479[M + H-C_7_H_11_]^+^	*α*-Linolenic Acid	WsAG < FgAG VIP: 5.24 *p* < 0.001	s
116	18.24	C_32_H_50_O_4_	498.3722	498.3709	2.4	521.3614[M+Na]^+^, 484.3365[M + H-CH_3_]^+^, 439.3322[M + H-C_2_H_3_O_2_]^+^, 303.3080[M-C_12_H_20_O_2_]^+^, 263.2783[M-C_15_H_24_O_2_]^+^, 248.2610[M + H-C_16_H_26_O_2_]^+^, 203.0991[M + H-C_2_H_3_O_2_-C_15_H_22_O_2_]^+^	3-*O*-Acetyloleanolic acid	WsAG, FgAG	a
117 *	18.50	C_19_H_24_O_2_	284.1773	284.1776	−1.1	285.1843[M + H]^+^, 259.2243[M + H-C_2_H_2_]^+^, 243.1701[M + H-C_2_H_2_O]^+^, 159.1308[M + H-C_8_H_13_O]^+^, 122.1168[M + H-C_11_H_11_O]^+^	Androsta-1,4-diene-3,17-dione	WsAG > FgAG VIP: 6.16 *p* < 0.001	a
118	19.97	C_16_H_28_O_3_	268.2039	268.2038	0.4	291.1950[M+Na]^+^, 223.1536[M + H-HCOOH]^+^, 123.0441[M + H-H_2_O-C_7_H_13_O_2_]^+^, 95.0141[M + H-C_4_H_9_O-C_5_H_9_O_2_]	13-Hydroxy-9,11-hexadecanedioic acid	WsAG, FgAG	[34]
119	20.15	C_17_H_24_O_2_	260.1766	260.1776	−3.8	261.1839[M + H]^+^, 243.1708[M + H-H_2_O]^+^, 221.1479[M + H-CH_3_-C_2_H_3_]^+^, 159.0791[M + H-H_2_O-C_6_H_13_]^+^	Panaxydol	WsAG, FgAG	[68]
120	20.49	C_17_H_26_O_3_	280.3138	280.3130	3.3	303.3030[M+Na]^+^, 252.2401[M + H-C_2_H_5_]^+^, 149.1310[M + H-C_10_H_21_]^+^,140.1322[M + H-C_10_H_21_]^+^, 97.1025[M + H-C_13_H_27_]^+^	1-Eicosene	WsAG, FgAG	a
121	22.88	C_19_H_38_O_4_	330.2766	330.2770	−1.2	353.2658[M+Na]^+^, 313.2725[M + H-H_2_O]^+^, 280.2603[M + H-2H_2_O-CH_3_]^+^, 239.2352[M + H-C_3_H_7_O_3_]^+^, 99.0871[M + H-C_4_H_7_O_4_-C_8_H_17_]^+^	Monopalmitin	WsAG, FgAG	[30]

* Characteristic component in WsAG. ^#^ Characteristic component in FgAG. ^s^ Identified with a standard, ^a^ Compared with spectral data obtained from Wiley Subscription Services, Inc. (New York, NY, USA).

**Table 2:** Details of FgAG and WsAG samples.

Species and the Morphological Features	Source	Growth Year	Collection Time	Batch No.
FgAGs *Main roots 9~15 cm (length) × 1.5~3.0 cm (diameter); 2~3 branch roots with diameters of 2~3.5 cm; fibrous roots with diameters of 0.1~0.2 cm; 3~4 stem scars in rhizomes; no adventitious roots.*	Ji’an City, Jilin Province, China	3, 4	2017.09–2017.10	FgAG1, 11
	Fusong County, Jilin Province, China	3, 4	2017.09–2017.10	FgAG2, 12
	Tonghua City, Jilin Province, China	3, 4	2017.09–2017.10	FgAG3,13
	Jingyu Country, Jilin Province, China	3, 4	2017.09–2017.10	FgAG4, 14
	Antu Country, Jilin Province, China	3, 4	2017.09–2017.10	FgAG5, 15
	Hunchun City, Jilin Province, China	3, 4	2017.09–2017.10	FgAG6, 16
	Helong City, Jilin Province, China	3, 4	2017.09–2017.10	FgAG7, 17
	Huadian City, Jilin Province, China	3, 4	2017.09–2017.10	FgAG8, 18
	Huairou District, Beijing Province, China	3, 4	2017.10–2017.11	FgAG9, 19
	Wendeng Area, Shandong Province, China	3, 4	2017.10–2017.11	FgAG10, 20
WsAGs *Main roots 5.0~6.0 cm (length) × 1.5~2.0 cm (diameter); 2~3 branch roots with diameters of 0.5~0.9 cm; fibrous roots with diameters of 0.1~0.2 cm; 15~25 stem scars in rhizomes; adventitious roots with diameters of 0.5~0.8 cm*	Lawton Coumtry, Michigan State, American	>15	2017.09–2017.11	WsAG1, 3, 6
	Schoharie County, Catskill region, American	>15	2017.09–2017.10	WsAG2, 4, 8
	Monongalia County, West Virginia, American	>15	2017.10–2017.11	WsAG5, 7
